# Vitamin D Deficiency Strongly Predicts Adverse Medical Outcome Across Different Medical Inpatient Populations: Results From a Prospective Study

**DOI:** 10.1097/MD.0000000000003533

**Published:** 2016-05-13

**Authors:** Lena Graedel, Meret Merker, Susan Felder, Alexander Kutz, Sebastian Haubitz, Lukas Faessler, Martha Kaeslin, Andreas Huber, Beat Mueller, Philipp Schuetz

**Affiliations:** From the Medical University Department (LG, SF, AK, SH, LF, BM, PS), University of Basel, Kantonsspital Aarau, Aarau; Medical Faculty of the University of Basel (MM), Basel; and Department of Laboratory Medicine (MK, AH), Kantonsspital Aarau, Aarau, Switzerland.

## Abstract

Vitamin D deficiency has been associated with several adverse outcomes mainly in the outpatient setting. The objective of this study was to examine the prevalence of vitamin D deficiency and its association with risk of adverse clinical outcomes in a large prospective cohort of medical inpatients.

We collected clinical data and measured 25(OH)D levels in adult medical patients upon hospital admission and followed them for 30 days. Regression analyses adjusted for age, gender, comorbidities, and main medical diagnosis were performed to study the effect of vitamin D deficiency on several hospital outcomes.

Of 4257 included patients, 1510 (35.47%) had 25(OH)D levels of 25 to 50 nmol/L (vitamin D insufficiency) and 797 (18.72%) had levels of <25nmol/L (severe deficiency). Vitamin D insufficiency and severe deficiency were associated (OR/HR, 95%CI) with an increased risk of 30-day mortality (OR 1.70, 1.22–2.36 and 2.70, 1.22–2.36) and increased length of stay (HR 0.88, 0.81–0.97 and 0.72, 0.65–0.81). Severe deficiency was associated with risk of falls (OR 1.77, 1.18–2.63), impaired Barthel index (OR 1.80, 1.42–2.28), and impairment in quality of life. Most associations remained robust after multivariate adjustment and in subgroups stratified by gender, age, comorbidities, and main diagnoses (*P* for interaction >0.05).

In this comprehensive and large medical inpatient cohort, vitamin D deficiency was highly prevalent and strongly associated with adverse clinical outcome. Interventional research is urgently needed to prove the effect of vitamin D supplementation on these outcomes.

## Abstract 2

Supplemental Digital Content is available in the text

## INTRODUCTION

In humans, vitamin D is acquired from transformation of 7-dehydrocholesterol in the epidermis by exposure to ultraviolet B radiation, as well as dietary intake. The vitamin is then metabolized in the liver to 25-hydroxyvitamin D (25(OH)D) and in the kidneys to the active 1,25-dihydroxyvitamin D (1,25(OH)2D). It has pleiotropic effects since a majority of cells in the human body express vitamin D receptors. 1,25(OH)2D may interact with over 200 genes thereby influencing cellular proliferation, differentiation, apoptosis, angiogenesis, insulin and renin production, bone and muscle metabolism, and stimulating macrophage cathelicidin production.^1–4^ In accordance with these cellular effects, several clinical studies reported negative effects of vitamin D deficiency on mortality risk,^5–8^ cancer,^9^ cardiovascular,^10^ and infectious diseases^11–13^ among others.

Although there is still controversy about optimal levels of vitamin D, a 25(OH)D serum concentration of less than 50 nmol/L is generally considered to indicate vitamin D insufficiency with levels of <25nmol/L indicating severe deficiency. To date, a sufficient vitamin D status is mostly defined by its benefits for the prevention of bone diseases. Optimal levels, however, may also depend on the patient population and medical illness with recent evidence suggesting that higher levels (up to 75–110 nmol/L) may positively influence medical diagnosis such as cancer or cardiovascular diseases.^14–18^

As vitamin D deficiency is increasingly being prevalent worldwide^19^ with a prevalence of about 50% in the healthy elderly population in Switzerland^20^ in-depth knowledge of this contributing factor to disease has important public health implications.^21^ Most of this research, however, has focused on outpatients and clinical data on medical inhospital patients are still scarce.

To close this gap, the aim of this prospective patient cohort study was to examine the associations of vitamin D deficiency and different adverse clinical outcomes within different medical patient subgroups.

## METHODS

### Study Design and Setting

This is an observational, prospective cohort study. Between March 2013 and February 2014 consecutive adult medical patients were included upon hospital admission in the emergency department into the quality-control TRIAGE project. This project's main aim is to optimize the triage and patient flow of adult patients with medical emergencies.^22^

As an observational quality control study, the Institutional review board of the Canton of Aargau has approved the study and waived the need for informed consent (EK 2012/059).

### Patient Population and Management

We included consecutive adult patients with an acute medical illness seeking inhospital care. Surgical and pediatric patients were excluded. Initial vital signs and other clinical information such as socio-demographics, main medical diagnosis, and comorbidities were recorded upon admission. Left over blood samples of all patients were collected and stored for later analyses including measurement of vitamin D levels. Clinical information and patient outcomes were assessed until hospital discharge and all patients were contacted by a structured phone interview 30 days after admission in order to evaluate satisfaction with care, clinical and functional outcome measures, quality of life measures, performance in activities of daily living, as well as rehospitalization rates and mortality. In case the patient could not be reached, family members or the general practitioner were contacted.^22^

### Main Diagnosis and Comorbidities

Patients were divided according to their leading diagnosis including infections, cardiovascular diseases, metabolic diseases, cancer, neurological disorders, digestive tract diseases, pulmonary diseases, and other disease. Metabolic diseases thereby included different diseases such as diabetes with hypoglycemia or hyperglycemia, hyperlipidemia, and severe electrolyte disturbances among others. We also defined the following comorbidity groups: congestive heart failure, chronic obstructive pulmonary disease, dementia, diabetes mellitus, cancer, and chronic renal failure. Nutritional status was assessed using the Nutritional Risk Screening 2002 within 48 hours after hospital admission. A risk of malnutrition was defined as a Nutritional Risk Screening 2002 score of ≥3 points.^23^

### Outcome

Our primary outcome was 30 day all-cause mortality assessed during the hospital stay and by telephone interviews at day 30.

Secondary outcomes included functional impairment, quality of life, length of hospital stay (LOS), readmission rate, and falls and fractures. Performance of daily living was measured by the Barthel index.^24^ We defined functional impairment as a Barthel index < 95 points. In order to assess quality of life, we used the standardized measure of health questionnaire EQ-5D including a descriptive system with 5 dimensions (mobility, self-care, usual activities, pain/discomfort, and anxiety/depression), as well as a Europe specific summary index value.^25^ These results were displayed as 2 levels, “impairments” or “no impairments.” The occurrence of falls and fractures was registered during hospitalization and in the 30 days following hospitalization by telephone interview.

### Assessment of Vitamin D Levels and Definition of Insufficiency

25(OH)D levels were measured on left-over samples upon admission using a chemiluminescence immunoassay for the quantitative determination of vitamin D. This method determines 25(OH)D3 as well as 25(OH)D2. We defined a normal vitamin D status as serum 25(OH)D > 50 nmol/L (>20 ng/mL), vitamin D insufficiency as 25 to 50 nmol/L (10–20ng/mL), and a severe deficiency as <25nmol/L (<10 ng/mL).^26^

### Statistical Analysis

Categorical variables are expressed as percentages and counts or vice versa and continuous variables as medians (interquartile ranges, 25th–75th percentiles), unless stated otherwise. Frequency comparison was done by the Chi-square test. For all binary endpoints logistic models with odds ratios (ORs) and 95% confidence intervals (95%CIs) were used. For time to hospital discharge, Cox regression models with hazard ratios were calculated. To adjust for possible confounding we used 3 statistical models: model 1 for age and gender; model 2 for age, gender, and comorbidities; model 3 for age, gender, comorbidities, and main diagnosis.

We evaluated the association between vitamin D levels and outcomes in the overall population as well as within different predefined subgroups based on gender, age (cut-off 75 years), and main medical diagnosis. Evidence of effect modification within these subgroups was assessed by inclusion of interaction terms into the statistical models. A *P* value <0.05 (for a 2-sided test) was considered statistically significant. All statistical analyses were performed with STATA 12.1 (Stata corp, College Station, TX).

## RESULTS

### Patient Population

Of 4257 included patients, 1510 (35.47%) had 25(OH)D levels of 25 to 50 nmol/L (vitamin D insufficiency) and 797 (18.72%) had levels of <25nmol/L (severe deficiency). Baseline characteristics of the overall population stratified according to vitamin D status are summarized in Table 1. Patients with an inadequate vitamin D level were older and were at higher risk of malnutrition. Laboratory analysis in patients with low vitamin D levels revealed lower serum albumin levels, lower albumin corrected serum calcium levels and higher serum levels of creatinine. Also, patients being included during winter and spring season had significantly lower vitamin D levels as compared to summer and fall.

**TABLE 1:**
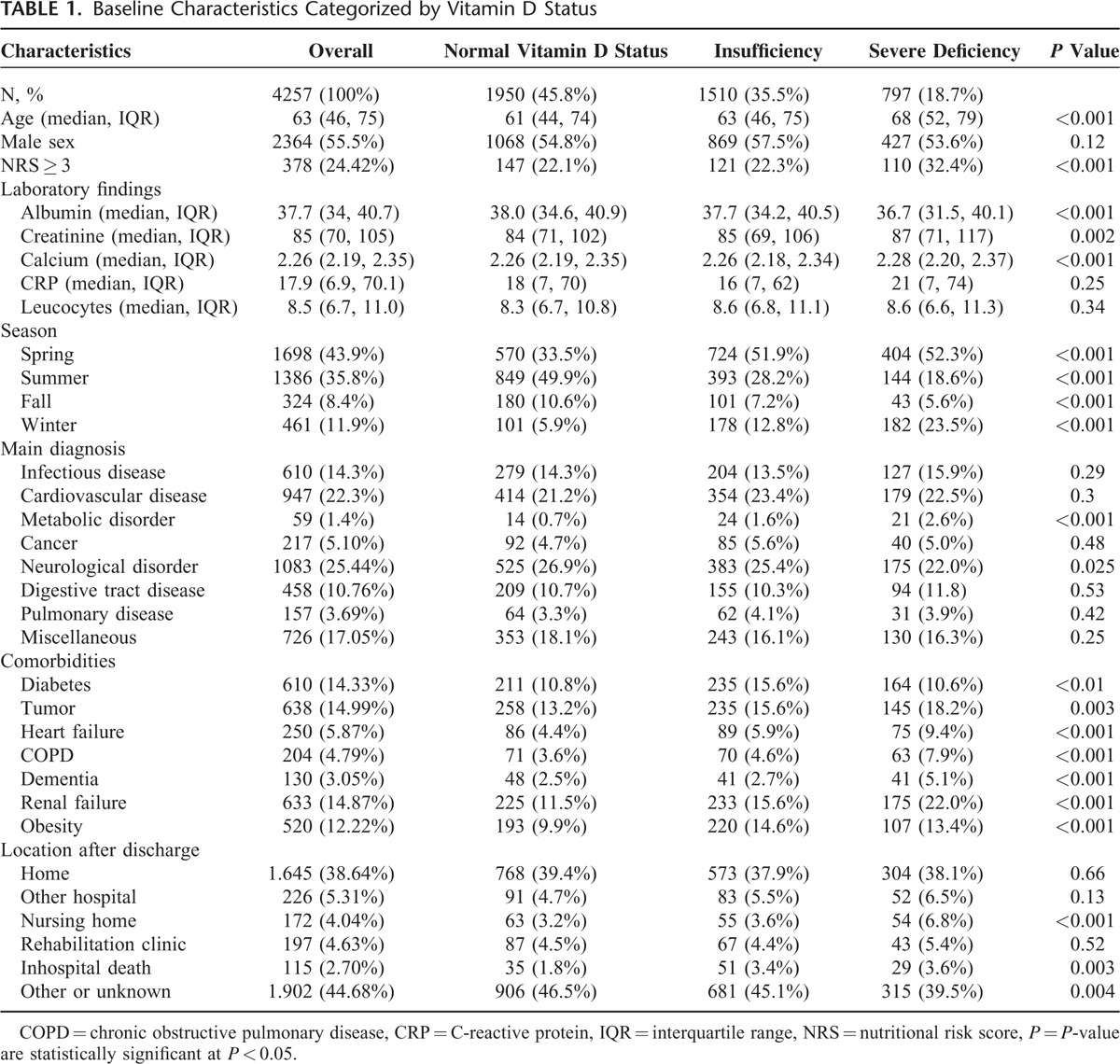
Baseline Characteristics Categorized by Vitamin D Status

### Primary Endpoint: Association of Vitamin D and Mortality

With lower vitamin D levels we found a stepwise increase in mortality of 3.4%, 5.6%, and 8.7% in patients with vitamin D sufficiency, insufficiency, and severe deficiency, respectively. In regression analysis, the unadjusted OR of 30-day mortality for vitamin D insufficient patients compared to sufficient levels (reference) was 1.70 (95%CI 1.22–2.36, *P* = 0.002) and in severely deficient patients 2.70 (1.22–2.36, *P* < 0.001). After stepwise adjustment for demographics (model 1), comorbidities (model 2), and medical diagnosis (model 3), the associations remained significant with an OR of 1.49 (95%CI 1.03–2.14, *P* = 0.03) for insufficient and 1.92 (95%CI 1.29–2.14, *P* = 0.001) for severely deficient patients in the fully adjusted model (for detailed results see Table 2).

**TABLE 2:**
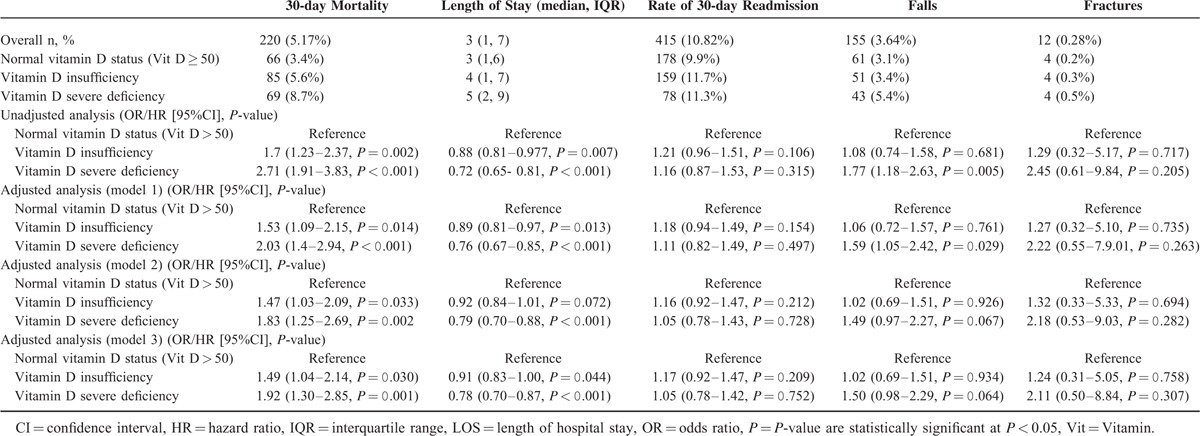
Association of Vitamin D Insufficiency (25–50 nmol/L) and Severe Deficiency (<25 nmol/L) and Adverse Medical Outcomes (30-day Mortality, LOS, 30-day Readmission Rate, Falls, and Fractures) Adjusted for Age and Gender (Model 1), Age, Gender, and Comorbidities (Model 2), and Age, Gender, Comorbidities, and Main Diagnosis (Model 3)

**FIGURE 1:**
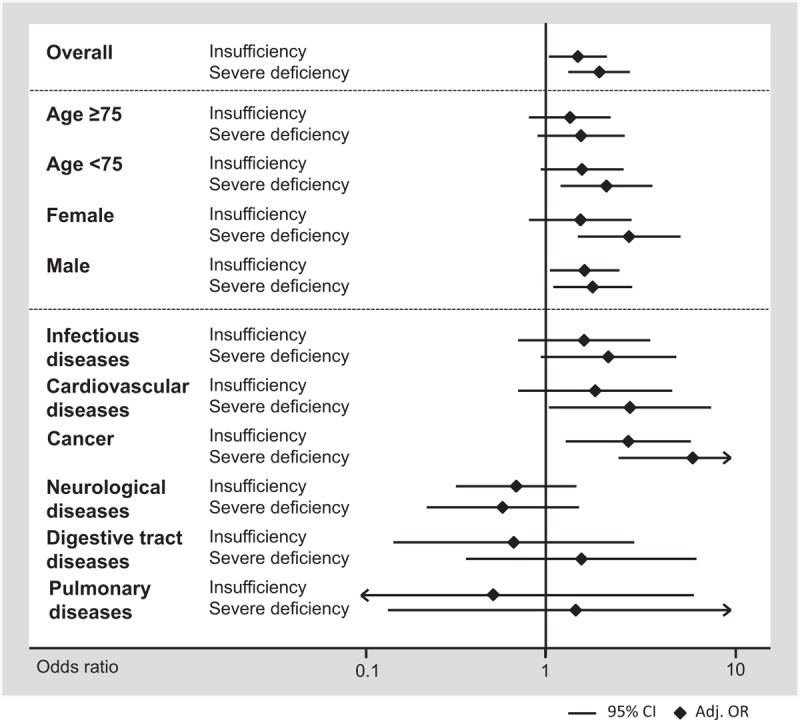
Predictive value of vitamin D insufficiency and deficiency for 30-day mortality by patient subgroups.

We also investigated whether the association of vitamin D deficiency and 30 day mortality would differ among subgroups of different demographics and main diagnoses (Appendix 1 and in Figure 1). For most analyses, results were robust and no evidence of effect modification (*P* > 0.05) was found, except for neurological disorders, for which the associations became weaker.

### Secondary Endpoints: Vitamin D and Hospital Outcomes

LOS was stepwise increased in vitamin D sufficient, insufficient, and severely deficient patients (3 [1, 6], 4 [1, 7], and 5 [2, 9] days, respectively, *P* < 0.001).

**FIGURE 2:**
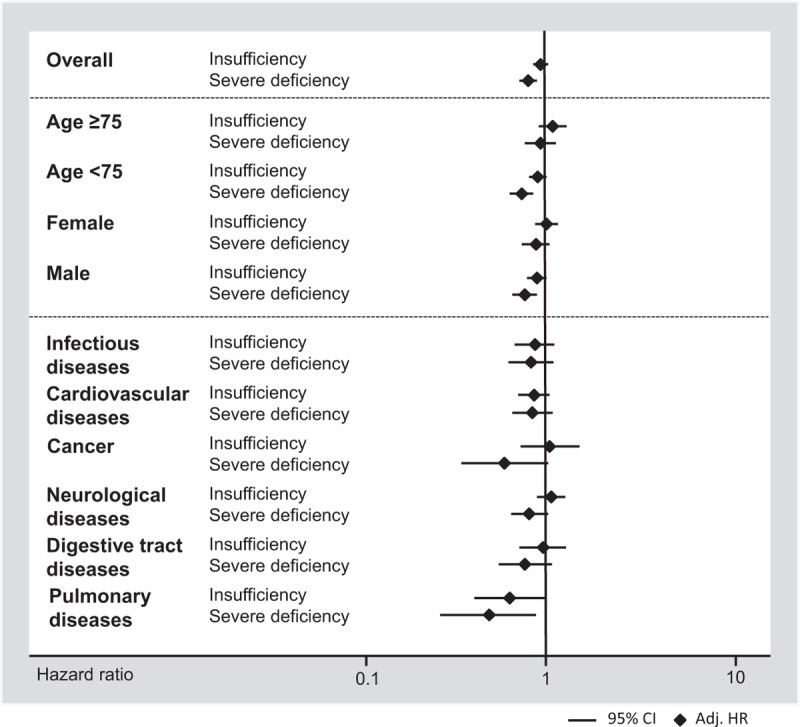
Predictive value of vitamin D insufficiency and deficiency for time to hospital discharge by patient subgroups.

**FIGURE 3:**
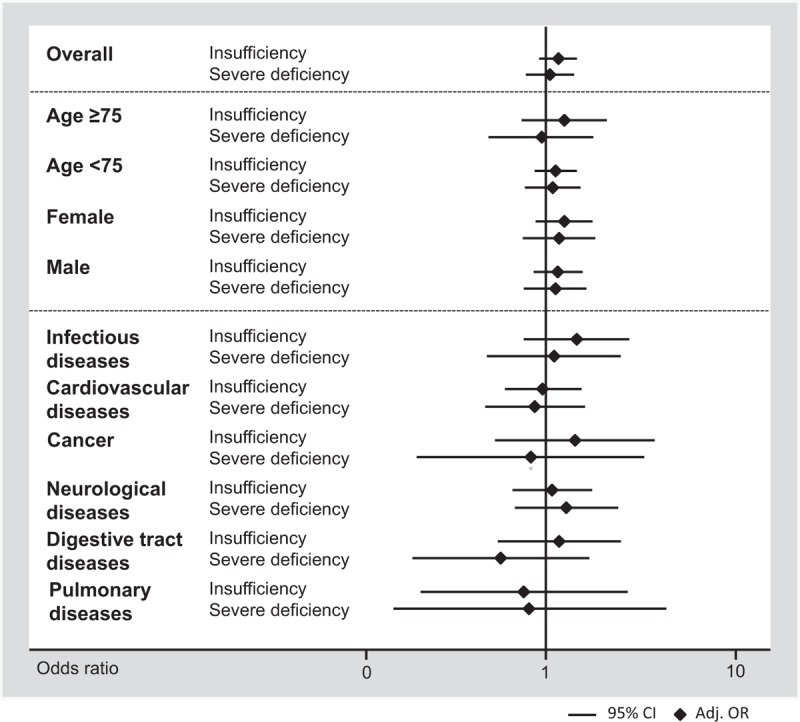
Predictive value of vitamin D insufficiency and deficiency for 30-day readmission rate by patient subgroups.

Unadjusted hazard ratio for time to hospital discharge was 0.88 (0.81–0.97, *P* = 0.007) and 0.72 (0.65–0.81, *P* < .001). These associations remained mostly significant after multivariate adjustment (Table 3). Similarly, we found a higher frequency in impairment in activities of daily living (Barthel index < 95 points) based on vitamin D levels, with 11.3%, 11.6%, and 18.7% in vitamin D sufficient, insufficient, and severely deficient patients, respectively. For severely deficient patients the association was significant with an OR of 1.80 (95%CI 1.42–2.28, *P* < 0.001), which remained robust after adjustment. In subgroup analysis (Appendix 1 and Figures 2 and 3), there was evidence of effect modification (*P* < 0.05) with the main diagnosis of cardiovascular diseases, which had an even stronger association. For hospital readmission, we found no association of low vitamin D levels with 30-day hospital readmission rate overall, and in different subgroups (no effect modifications).

**TABLE 3:**
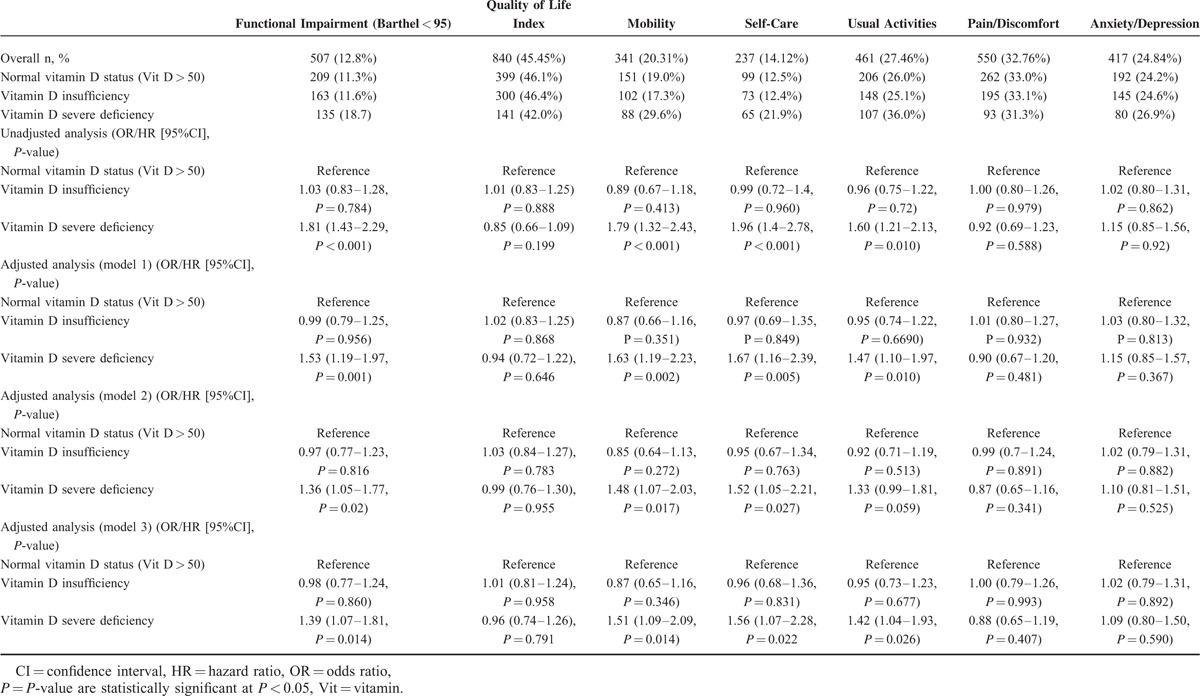
Association of Vitamin D Insufficiency (25–50 nmol/L) and Severe Deficiency (<25 nmol/L) and Adverse Medical Outcomes (Functional Impairment, Quality of Life) Adjusted for Age and Gender (Model 1), Age, Gender, and Comorbidities (Model 2), and Age, Gender, Comorbidities, and Main Diagnosis (Model 3)

### Falls/Fractures and Quality of Life in the Follow-Up

We found a stepwise increase in reports of falls in the vitamin D deficient and severely deficient patients (3.1%, 3.4%, and 5.4%), yet after adjustment these associations did not remain significant. Also, the frequency of reported fractures did not depend on vitamin D status (*P* = 0.69).

**FIGURE 4:**
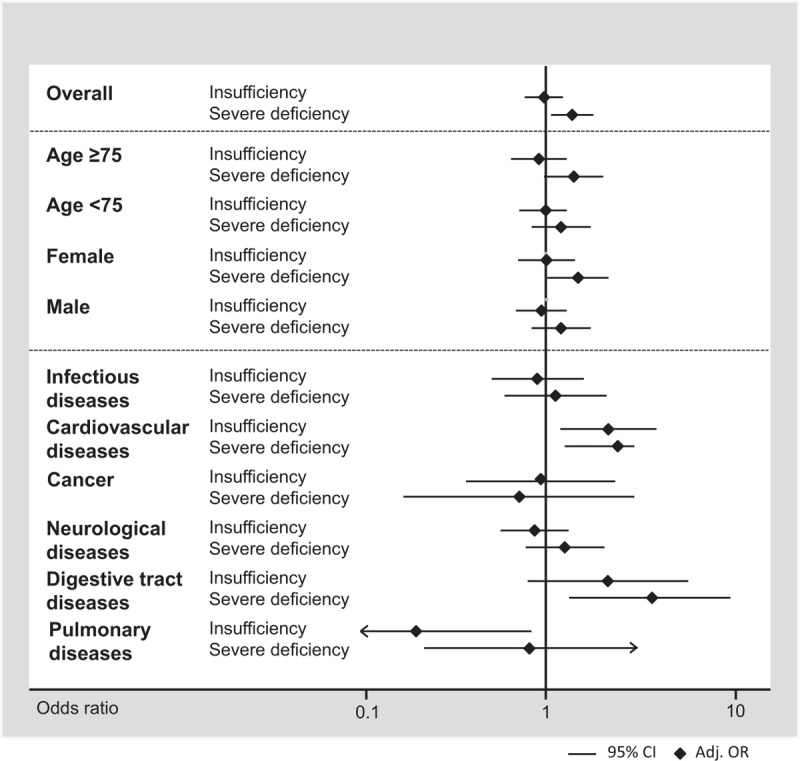
Predictive value of vitamin D insufficiency and deficiency for functional impairment by patient subgroups as measured by the Barthel index.

**FIGURE 5:**
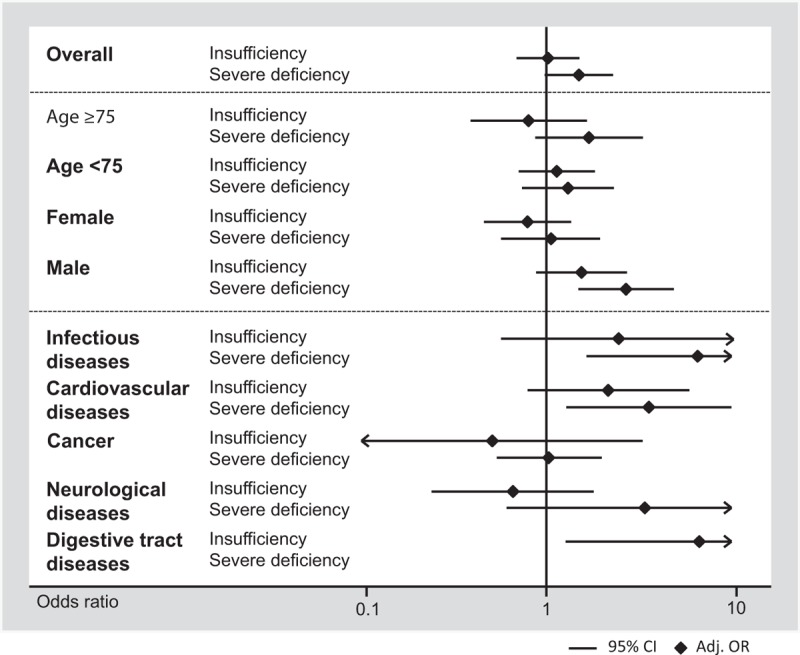
Predictive value of vitamin D insufficiency and deficiency for falls by patient subgroup.

We also observed an increase in reported impairment in quality of life (defined by mobility, self-care, usual activity, pain/discomfort, and anxiety/depression) in patients with a severe vitamin D deficiency compared to patients with sufficient vitamin D status, except for the dimensions pain/discomfort and anxiety/depression. The association between severe vitamin D deficiency and decreased quality of life remained significant after adjustment save for pain/discomfort and anxiety/depression (model 1–3) and usual activities in model 2. In subgroup analyses (Appendix 1 and Figures 4 and 5), there was no evidence of effect modification (*P* > 0.05).

## DISCUSSION

This findings of our study including a large and well-characterized cohort of medical inpatients with different diseases with vitamin D levels measured upon hospital admission are 3-fold: 1st, we found a high prevalence of >50% of vitamin D deficiency with levels below 50 nmol/L and almost 20% of patients had with severe deficiency with levels below 25 nmol/L. This accords with findings from a previous study by Thomas et al^27^ as well as data from the Federal Office of Public health in Switzerland.^28^ Second, we found strong associations of vitamin D deficiency and several patient relevant adverse clinical outcomes measured during the hospital stay and after 30 days including mortality, LOS, falls and functional impairment, and quality of life. Third, most of these associations remained robust after multivariate adjustment and within different subgroups suggesting that effects are rather not disease specific but more general for medical inpatients.

Several clinical studies have been conducted investigating association of vitamin D deficiency and different adverse medical outcomes for medical patients, such as mortality,^29–42^ LOS,^36,38,43–45^ functional impairment,^46,47^ higher disease severity,^39–41,48^ and increased risk or severity of infections.^29,30,33,43,49–52^ However, most of these studies were small and limited to specific patient subgroups. Also, rigorous multivariate adjustment for possible confounders was not done in most studies. Although several studies suggest a link between vitamin D deficiency and various adverse outcomes, definite conclusions could not be drawn. To our knowledge, our study is first to examine associations of vitamin D deficiency and adverse outcome in a large and unselected patient population with multiple short-term medical outcomes and is thus able to close this gap.

However, our study is not able to prove causality and to answer the question whether vitamin D deficiency contributes to worse patient outcome or whether it is merely a surrogate of increased disease severity, lower exposure to sun, and/or malnutrition. To address this issue we used multivariate regression analysis including different potential confounders such as age and disease. Most of the observed associations were robust. Still, interventional research is needed to answer the question whether vitamin D supplementation in the acute phase of illness in medical inpatients has beneficial effects on outcome.

Vitamin D is also considered as a negative acute-phase protein, which could explain the observed low levels during acute illness and the association between low levels and adverse outcome such as higher mortality. Several mechanisms may be responsible for such as a decrease of vitamin D carrier proteins, increased conversion of 25(OH)D to 1,25(OH)2D, as well as hemodilution.^53,54^ Similar to other studies, we also found significantly lower albumin level in vitamin D deficient patients.^55,56^ Thus, measuring total serum 25(OH)D may not be adequate to assess vitamin D status during acute illness. The free fraction of 25(OH)D may be a more accurate representation, because it is not influenced by albumin levels and it represents the physiologically important fraction. However, measurement of the bioavailable fraction is technically more challenging.^55,57^

Vitamin D has also been suggested to have antiinflammatory and antiproliferative properties.^4,58^ These effects may physiopathologically explain the increase in adverse outcomes with deficient vitamin D status and call for the substitution of patients in order to correct impaired vitamin D status.^59^ However, without interventional data in this patient population such conclusions may not be warranted. Current recommendations for vitamin D screening and supplementation are based on data regarding the benefits for musculoskeletal outcomes. Many large observational studies have been conducted on the benefits of vitamin D supplementation on falls and fractures.^60,61^ Still, even for musculoskeletal health there is remaining ambiguity with recent randomized trials reporting conflicting results.^62,63^

The study has several strengths: we studied a large, well-defined cohort with a heterogeneous patient population, thus justifying the validity of our data in the general population. In order to assess impairments in quality of life and activities of daily living we used standardized tools, which furthermore facilitate the comparison of our results. Vitamin D levels were measured in a consecutive sample limiting the risk of selection bias. Nevertheless, some limitations of our study should be considered. It was a single-center study including Swiss patients and results may not be generalizable to other countries. Although we adjusted for potential confounders, we cannot eliminate the possibility of residual confounding factors influencing our results. For example, parathyroid hormone levels were not available. Also, there is controversy regarding the best methodology for measuring 25(OH)D. High-performance liquid chromatography is generally considered the gold-standard, as it is more accurate and reliable than the chemiluminescence immunoassay we used in our study. However, this method is time and labor intensive and requires expensive equipment, thus making it unsuitable to high-capacity clinical laboratories.^64,65^ In addition, we did not have data available on chronicity of disease which may be an important factor explaining the relationship of low vitamin D levels and adverse outcome. Most importantly, as an observational study it is more hypothesis generating and does not address causality.

## CONCLUSION

Our study confirms results from previous studies and shows a high prevalence of inadequate vitamin D status in the medical inpatient population with strong associations with different adverse medical outcomes and robust results among different medical diagnosis subgroups. High-quality randomized controlled studies are needed to assess whether vitamin D supplementation could improve the course and recovery from an acute illness in the medical inpatient setting.

## Supplementary Material

Supplemental Digital Content
