# Supplementary material for: Vitamin D Deficiency Strongly Predicts Adverse Medical Outcome Across Different Medical Inpatient Populations: Results From a Prospective Study
Source: Medicine (Baltimore). 2016 May 13;95(19):e3533. doi: 10.1097/MD.0000000000003533 (PMC4902492; doi:10.1097/MD.0000000000003533)
Supplement: Supplemental Digital Content [file medi-95-e3533-s001.docx]

| **Appendix 1: Subgroup Analysis** | | | | |
| --- | --- | --- | --- | --- |
| **OUTCOME** |  | **Insufficiency** OR (95%CI), p-value | **Severe deficiency** OR (95%CI), p-value | p-value* |
| **30-day Mortality** | **Overall (%)** | 1.49 (1.04-2.14), .03 | 1.92 (1.3-2.85), .001 |  |
|  | **Age** |  |  |  |
|  | ≥75 | 1.37 (0.82-2.26), .227 | 1.55 (0.90-2.67), .115 | .227 |
|  | <75 | 1.57 (0.94-2.63), .082 | 2.13 (1.20-3.78), .01 |  |
|  | **Gender** |  |  |  |
|  | Male | 1.62 (1.05-2.50), .029 | 1.79 (1.10-2.93), .02 | .852 |
|  | Female | 1.54 (0.81-2.91), .188 | 2.82 (1.49-5.35), .001 |  |
|  | **Medical diagnosis** |  |  |  |
|  | Infectious diseases | 1.61 (0.71-3.67), .258 | 2.18 (0.94-5.08), .07 | .789 |
|  | Cardiovascular diseases | 1.85 (0.71-4.82), .208 | 2.85 (1.04-7.83), .042 | .591 |
|  | Cancer | 2.79 (1.28-6.08), .01 | 6.24 (2.47-15.75), <.001 | **.078** |
|  | Neurological disorder | 0.70 (0.33-1.48), .347 | 0.59 (0.23-1.53), .279 | **.043** |
|  | Digestive tract diseases | 0.67 (0.15-3.01), .602 | 1.56 (0.37-6.53), .545 | .421 |
|  | Pulmonary diseases | 0.52 (0.04-6.32), .607 | 1.45 (0.14-14.97), .757 | .268 |
|  |  |  |  |  |
| **Rehospitalisation** | **Overall (%)** | 1.17 (0.92-1.47), .209 | 1.05 (0.78-1.42), .752 |  |
|  | **Age** |  |  |  |
|  | ≥75 | 1.26 (0.74-2.14), .399 | 0.95 (0.49-1.81), .866 | .895 |
|  | <75 | 1.13 (0.87-1.47), .362 | 1.09 (0.77-1.54), .626 |  |
|  | **Gender** |  |  |  |
|  | Male | 1.16 (0.86-1.58), .327 | 1.13 (0.76-1.66), .549 | .943 |
|  | Female | 1.26 (0.88-1.79), .205 | 1.18 (0.75-1.85), .474 |  |
|  | **Medical diagnosis** |  |  |  |
|  | Infectious diseases | 1.47 (0.76-2.83), .25 | 1.11 (0.48-2.54), .808 | .794 |
|  | Cardiovascular diseases | 0.96 (0.60-1.56), .881 | 0.87 (0.47-1.63), .67 | .456 |
|  | Cancer | 1.44 (0.53-3.88), .476 | 0.83 (0.20-3.41), .791 | .811 |
|  | Neurological disorder | 1.08 (0.66-1.78), .763 | 1.29 (0.68-2.46), .438 | .743 |
|  | Digestive tract diseases | 1.18 (0.55-2.55), .674 | 0.57 (0.19-1.72), .316 | .379 |
|  | Pulmonary diseases | 0.76 (0.21-2.77), .676 | 0.81 (0.15-4.48), .807 | .937 |
|  |  |  |  |  |
| **Falls** | **Overall (%)** | 1.02 (0.69-1.51), .934 | 1.5 (0.98-2.29), .064 |  |
|  | **Age** |  |  |  |
|  | ≥75 | 0.80 (0.39-1.66), .552 | 1.70 (0.87-3.33), .124 | .2 |
|  | <75 | 1.14 (0.71-1.83), .581 | 1.31 (0.74-2.32), .351 |  |
|  | **Gender** |  |  |  |
|  | Male | 1.55 (0.88-2.73), .13 | 2.70 (1.49-4.89), .001 | .237 |
|  | Female | 0.79 (0.46-1.36), .391 | 1.06 (0.57-1.95), .854 |  |
|  | **Medical diagnosis** |  |  |  |
|  | Infectious diseases | 2.46 (0.57-10.63), .228 | 6.58 (1.65-26.21), .008 | .545 |
|  | Cardiovascular diseases | 2.16 (0.79-5.93), .136 | 3.58 (1.28-10.00), .015 | **.006** |
|  | Cancer | 0.51 (0.07-3.32), .477 | 1 | **.02** |
|  | Neurological disorder | 1.03 (0.54-1.99), .921 | 0.66 (0.24-1.80), .414 | .188 |
|  | Digestive tract diseases | 3.41 (0.61-18.93), .161 | 6.71 (1.27-35.53), .025 | **.051** |
|  | Pulmonary diseases | 1 | 1 | .356 |
|  |  |  |  |  |
| **Functional impairment** | **Overall (%)** | 0.98 (0.77-1.24), .86 | 1.39 (1.07-1.81), .014 |  |
|  | **Age** |  |  |  |
|  | ≥75 | 0.92 (0.65-1.30), .637 | 1.42 (0.98-2.05), .062 | .868 |
|  | <75 | 1.0 (0.72-1.38), .999 | 1.35 (0.92-1.99), .124 |  |
|  | **Gender** |  |  |  |
|  | Male | 0.95 (0.69-1.30), .745 | 1.21 (0.84-1.75), .312 | .96 |
|  | Female | 1.00 (0.71-1.44), .959 | 1.50 (1.02-2.19), .037 |  |
|  | **Medical diagnosis** |  |  |  |
|  | Infectious diseases | 0.90 (0.51-1.61), .736 | 1.13 (0.60-2.14), .697 | .098 |
|  | Cardiovascular diseases | 2.18 (1.20-3.98), .011 | 2.46 (1.27-4.78), .008 | **.009** |
|  | Cancer | 0.94 (0.37-2.38), .894 | 0.72 (0.17-3.01), .652 | .233 |
|  | Neurological disorder | 0.87 (0.57-1.33), .523 | 1.27 (0.78-2.08), .338 | .652 |
|  | Digestive tract diseases | 2.17 (0.80-5.91), .13 | 3.76 (1.34-10.54), .012 | .316 |
|  | Pulmonary diseases | 0.20 (0.05-0.84), .029 | 0.83 (0.22-3.07), .78 | .316 |
|  |  |  |  |  |
| **Impairment in quality of life** | **Overall (%)** | 1.00 (0.80-1.23), .958 | 1.04 (0.79-1.36), .791 |  |
|  | **Age** |  |  |  |
|  | ≥75 | 1.03 (0.69-1.52), .893 | 1.02 (0.66-1.58), .931 | .809 |
|  | <75 | 0.97 (0.75-1.26), .779 | 1.05 (0.74-1.49), .779 |  |
|  | **Gender** |  |  |  |
|  | Male | 0.89 (0.63-1.24), .409 | 1.19 (0.78-1.82), .78 | .998 |
|  | Female | 1.08 (0.82-1.43), .573 | 0.92 (0.64-1.33), .665 |  |
|  | **Medical diagnosis** |  |  |  |
|  | Infectious diseases | 1.25 (0.70-2.24), .196 | 1.64 (0.77-3.47), .196 | .878 |
|  | Cardiovascular diseases | 1.26 (0.80-1.98), .314 | 0.88 (0.48-1.59), .666 | .255 |
|  | Cancer | 0.71 (0.27-1.84), .482 | 0.66 (0.15-2.83), .578 | .651 |
|  | Neurological disorder | 1.13 (0.76-1.69), .531 | 1.36 (0.78-2.36), .275 | **.098** |
|  | Digestive tract diseases | 1.06 (0.51-2.19), .877 | 1.35 (0.59-3.08), .476 | .967 |
|  | Pulmonary diseases | 0.43 (0.15-1.19), .105 | 0.96 (0.27-3.36), .944 | .606 |
| **OUTCOME** |  | HR (95%CI), p-value | HR (95%CI), p-value | p-value* |
| **Time to hospital discharge** | **Overall (%)** | 0.91 (0.83-1.00), .044 | 0.78 (0.70-0.87), <.001 |  |
|  | **Age** |  |  |  |
|  | ≥75 | 1.06 (0.89-1.26), .517 | 0.91 (0.75-1.10), .311 | .487 |
|  | <75 | 0.88 (0.79-0.98), .022 | 0.72 (0.62-0.83), <.001 |  |
|  | **Gender** |  |  |  |
|  | Male | 0.87 (0.77-0.98), .022 | 0.75 (0.64-0.87), <.001 | .207 |
|  | Female | 0.98 (0.85-1.13), .796 | 0.86 (0.72-1.02), .078 |  |
|  | **Medical diagnosis** |  |  |  |
|  | Infectious diseases | 0.85 (0.66-1.08), .187 | 0.81 (0.61-1.07), .136 | .964 |
|  | Cardiovascular diseases | 0.84 (0.69-1.02), .079 | 0.82 (0.64-1.06), .128 | .439 |
|  | Cancer | 1.02 (0.71-1.48), .906 | 0.58 (0.34-1.00), .048 | .378 |
|  | Neurological disorder | 1.04 (0.87-1.24), .69 | 0.79 (0.63-1.00), .049 | .903 |
|  | Digestive tract diseases | 0.94 (0.70-1.25), .659 | 0.75 (0.54-1.05), .097 | .713 |
|  | Pulmonary diseases | 0.62 (0.40-0.96), .033 | 0.48 (0.26-0.86), .013 | .107 |
|  |  |  |  |  |
| *p-value: p-value for effect modification; HR, hazard ratio; OR, Odds ratio; p, p-value are statistically significant at p<0.05 | | | | |
